# *In silico* functional elucidation of uncharacterized proteins of *Chlamydia abortus* strain LLG

**DOI:** 10.4155/fsoa-2016-0066

**Published:** 2017-01-24

**Authors:** Gagandeep Singh, Dixit Sharma, Vikram Singh, Jyoti Rani, Francessco Marotta, Manoj Kumar, Gorakh Mal, Birbal Singh

**Affiliations:** 1 Centre for Computational Biology & Bioinformatics, Central University of Himachal Pradesh, Shahpur 176206, India; 2 Department of Botany, Punjabi University, Patiala 147002, India; 3 ReGenera Research Group of Aging-Intervention & Montenapoleone Medical Centre, Milano, Italy; 4 Department of Microbiology & Immunology, National Institute of Nutrition, Hyderabad 500007, India; 5 ICAR-Indian Veterinary Research Institute, Regional Station, Palampur 176061, India

**Keywords:** *Chlamydia abortus*, functional annotation, hypothetical proteins

## Abstract

**Aim:**

This study reports structural modeling, molecular dynamics profiling of hypothetical proteins in *Chlamydia abortus* genome database.

**Methodology:**

The hypothetical protein sequences were extracted from *C. abortus* LLG Genome Database for functional elucidation using *in silico* methods.

**Results:**

Fifty-one proteins with their roles in defense, binding and transporting other biomolecules were unraveled. Forty-five proteins were found to be nonhomologous to proteins present in hosts infected by *C. abortus*. Of these, 31 proteins were related to virulence. The structural modeling of two proteins, first, WP_006344020.1 (phosphorylase) and second, WP_006344325.1 (chlamydial protease/proteasome-like activity factor) were accomplished. The conserved active sites necessary for the catalytic function were analyzed.

**Conclusion:**

The finally concluded proteins are envisioned as possible targets for developing drugs to curtail chlamydial infections, however, and should be validated by molecular biological methods.

## Lay abstract

Sequencing technologies have generated abundant data on genome and proteins of an organism. The hypothetical proteins are those whose existence is predicted by computational analysis of genes or protein sequences, but practical evidence to prove them are lacking. This study predicts functions of hypothetical proteins in *Chlamydia abortus* by computational and bioinformatics methods, determining their 3D structures by structural genomics, homology modeling with known proteins and annotating possible catalytic sites. These findings may be helpful for evolving strategies to curtail *Chlamydia abortus* infection.

## Graphical abstract

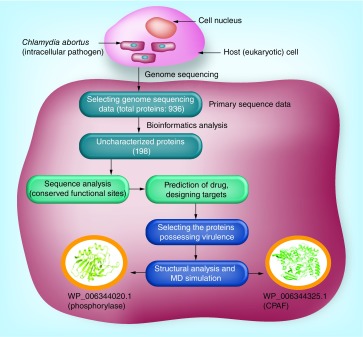

**Figure 1.**
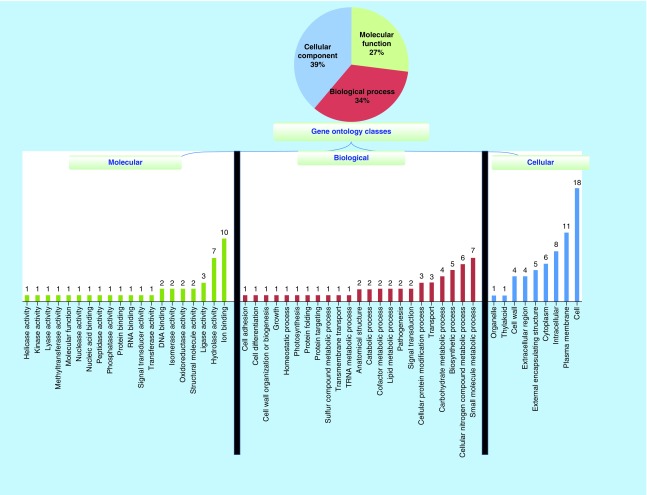
**Gene ontology of 51 hypothetical proteins in *Chlamydia abortus*.** The proteins are classified based on biological, cellular and molecular functions.

**Figure 2.**
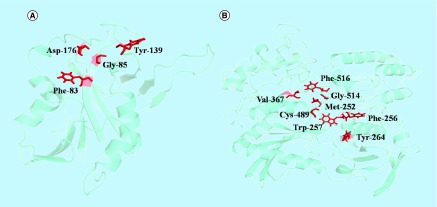
**Structural elucidation of two proteins from *Chlamydia abortus* genome sequence.** **(A)** WP_006344020.1 (phosphorylase) and **(B)** WP_006344325.1 (CPAF). The amino acid residues involved in enzymatic activity are shown in red.

**Figure 3.**
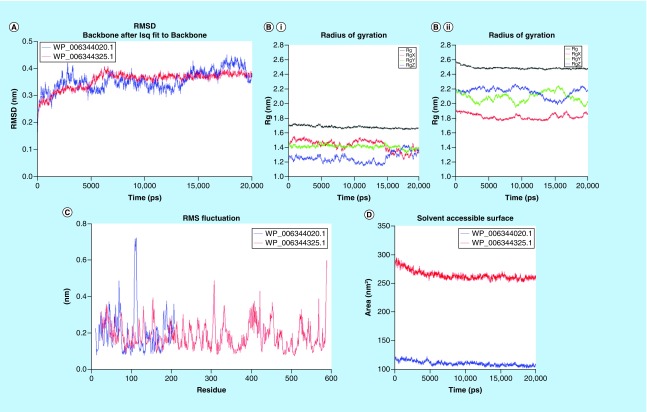
**Molecular dynamic analysis of two homology-modeled structures showed stability in the root-mean-square deviations and radius of gyration.** Also the structural flexibility of proteins were analyzed by RMSF of the amino acid residues. Steadiness of proteins were analyzed by SASA during the MD simulations that revealed that the proteins were stable. **(A)** RMSD of two homology-modeled proteins were plotted, initially both the proteins showed an increase in RMSD and after 2.5 ns, proteins attained stability and consistency in structural behavior, indicating the stability of proteins during MD simulation. **(B.I)** WP_006344020.1 and **(B.II)** WP_006344325.1. The proteins had initially less compactness in structures, but at later stages, the proteins showed more compactness and less variation in the radius of gyrations along all spatial axes. **(C)** Protein WP_006344020.1 (blue) and WP_006344325.1 (red) with amino acid residues, 198 and 565, respectively, showed evenness in the RMSF of amino acid residues of both the proteins with the progression of MD. The uniformity indicates that the proteins are stable. **(D)** The SASA of the two modeled proteins was constant throughout the MD simulations. MD: Molecular dynamics; RMSD: Root-mean-square deviations; RMSF: Root-mean-square fluctuations; SASA: Solvent surface accessible area.

*Chlamydia abortus* is an important, amphixenosis, nonmotile, Gram-negative, obligate intracellular pathogen [1,2]. The pathogen causes enzootic abortion, vesiculitis, orchitis and epididimytis in cattle [3]. When zoonotic, it causes conjunctivitis, health pathologies and abortion in sheep and goats [4–6], yaks [7], pig [8], cats [9], stray and companion animals [10]. The pathogen can be zoonotic spreading infection in humans. The infected people may not have outward symptoms in early stages, but *Chlamydia* can create serious health problems such as pelvic inflammatory disease in females. Incidences of psittacosis, primarily caused by *C. psittaci* have been noted in women involved in chicken gutting [11]. *C. abortus* has attracted increasing scientific attention due to its pathogenecity and severe systemic infection in humans as well some animals [12].

The bacterium is assigned to family *Chlamydiaceae* entailing two genera, namely *Chlamydia* and *Chlamydophila* comprising of nine species; three of *Chlamydia* (*C. muridarum, C. suis* and *C. trachomatis*), and six of *Chlamydophila* (*C. caviae, C. abortus, C. felis, C. pecorum, C. pneumoniae* and *C. psittaci*) [13]. As the genus, *Chlamydophila* is not widely accepted by mainstream research groups, the researchers have recommended reunifying the genera *Chlamydia* and *Chlamydophila* to one single genus, the genus *Chlamydia* within the family *Chlamydiaceae* [14,15].

The bacterial genome database has proteins classified as hypothetical, as their functions are not confirmed by molecular biological methods. The hypothetical proteins constitute around 20–40% of the total proteome, which are important for structural biologists. Their functions can be predicted by domain homology searches with various confidence levels. As wet lab methods, generally used for unraveling desired genes and proteins are expensive and time-consuming, the *in silico* methods have emerged as important tools to predict or identify the hypothetical genes and proteins. The genome of *C. abortus* LLG has also been sequenced and entails several hypothetical proteins [16]. This study reports functional analysis of unrecognized proteins from the genome data of *C. abortus* strain LLG. To the best of our knowledge, it is the first comprehensive description of unrecognized proteins in the species.

## Methodology

### Data extraction & identification of conserved signatures

The *C. abortus* LLG with genome reference number NZ_CM001168.1 at NCBI Genome database [17] served as data source. The sequences of unrecognized proteins were extracted from Genome Database at NCBI for functional inferences using *in silico* methods. Functional signature sequences of proteins were identified by Web-based tools, namely NCBI-CDD [18], INTERPROSCAN [19] and support vector machine (SVM)-Prot [20]. For predicting family and superfamily of the proteins, protein families (Pfam) [21] and structural classification of proteins (SCOP)-superfamily [22] were used. The conserved signature sequence and protein families were identified by these programs.

### Characterization & localization of proteins with conserved signatures

Computation of theoretical isoelectric point (pI) and molecular weight was determined by compute pI/molecular weight [23]. GRAVY CALCULATOR [24] was used for calculating the grand average of hydropathicity. Additionally, the properties of sequences including aromatic and aliphatic, basic and acidic sequences with average number of polar and nonpolar amino acids were determined by using EMBOOS PepStat [25]. To discover the position of these proteins with predicted signature sequences, TMHMM [26], HMMTOP [27] and SOSUI-GramN [28] were used, while subcellular localization of proteins was determined by CELLO [29,30]. The SignalP 4.1 [31] was used to determine peptide signal cleavage sites.

### Functional categorization & pathway analysis of proteins

Once signature sequence and proteins localization were identified, the functional roles of hypothetical proteins, and their gene ontology were predicted using CELLO2GO [32]. This program is used to predict the bacterial proteins at cellular, biological and molecular levels. To find the role of hypothetical proteins in various pathways, they were analyzed using KEGG database [33].

### Homology analysis of protein sequences with host proteins & virulence factor analysis

Basic Local Alignment Search Tool for proteins [34] was used for *C. abortus* hypothetical proteins against various hosts including *Ovis aries* (sheep; taxid: 9940), *Capra aegagrus hircus* (goat; taxid: 9925), *Bos taurus* (bovine; taxid: 9913), *Bos grunniens* (yak; taxid: 30521), *Sus scrofa domesticus* (pig; taxid: 9825) and *Homo sapiens* (human; taxid: 9906) proteins at NCBI database [35]. The sequences showing hits with less than 0.0001 expectation value were not considered, and concerned protein sequences were supposed to share homology with proteins in hosts [36]. Nonhomologous proteins were checked for virulence factors that are involved in severity of the infection, and are envisaged as targets for developing drugs against pathogen [37]. The virulence factor of nonhomologous proteins was identified by VICMpred and BTXpred [38,39]. Both methods for predicting virulence factor from protein sequence were based on SVM.

### Structural analysis & model evaluation of functionally identified proteins

Homology-modeling method, Phyre2 [40], was used for predicting the protein structure. The quality of model was recognized with similarity of sequence of target and the template approximation [34]. After prediction of structures, the models were validated for probable errors by using structure analysis and verification server (SAVES) program for molecular stereochemical quality, residues parameters, nonbonded interactions, model compatibility and macromolecular volume [41–44]. For residues in most favored region, the Ramachandran plot was analyzed in PROCHECK [45] at SAVES.

### Molecular dynamics simulation

Homology-modeled proteins were conceded for molecular dynamics (MD) simulations with GROMACS 5.0 (GROningen MAchine for Chemical Simulation) package using the GROMOS96 53a6 force field. To generate the topology files for proteins, the command pdb2gmx was used. Protein salvation was carried out, and the solvated proteins were positioned in a cubic box keeping a distance of 1.0 nm between the box edges and the protein surface. The particle-mesh ewald (PME) electrostatic and periodic boundary conditions were applied in the all directions [46]. Na^+^ counter ions were added to neutralize all the systems as per necessity in the proteins. 50,000 steps of steepest descent energy minimization were performed for all the systems to avoid high-energy interactions and steric clashes.

The equilibration and production phases are composition of MD simulation progression. The systems were administered to the simulations (constant number, volume and temperature [NVT] and constant number, pressure and temperature [NPT]) at 300 K for 100 ps to equilibrate the system. Ultimately, every system was subjected to MD production run at 1 bar pressure and 300 K temperature for 20 ns. The atom coordinates were recorded at every 10 ps throughout the MD simulation. Table 1 summarizes various bioinformatics programs used in this study.

## Results

### Data extraction & analysis of hypothetical proteins

The genome data of *C. abortus* LLG (NZ_CM001168.1 at NCBI Genome database; [17]) have a total of 936 proteins of which 198 proteins are termed as hypothetical. Of these, 51 proteins were predicted with conserved domains. The Pfam database revealed families of the identified conserved domains with their particular functions. A total of 37 sequences with particular family were identified. Additionally, superfamilies of 38 sequences were successfully determined. Identification of signature sequences and protein families enabled us to classify the hypothetical proteins in different categories including binding proteins, outer membrane proteins, enzymes, defense, secretory and signaling molecules. The supporting data have been shown in Supplementary Tables 1, 2 & 3. Additionally, re-analysis of whole proteome (including pre-characterized) was done, and no discrepancy between the official annotation and analysis by our methodology was observed (summarized in Supplementary Table 1).

### Localization of hypothetical proteins with conserved signatures

Out of 51 hypothetical proteins, 13 proteins were predicted to have transmembranic helices on the basis of TMHMM, HMMTOP and SOSUI-GramN. Furthermore, concerning subcellular localization, it was predicted that 32 proteins were cytoplasmic, three as extracellular, five as innermembranic, eight as outermembranic and three as periplasmic. Using SignalP 4.1, a total of seven proteins with cleavage sites were detected, of which only single peptide is found as transmembrane. The proteins predicted and their locations are summarized in Supplementary Table 3.

### Physiochemical characterization of proteins with functional domain

The sequence analysis for prediction of the functions showed a total of 51 proteins with conserved domain, gene IDs and some specific functions (Table 2). Of these, a total of 16 proteins were found to have theoretical pI equal to or more than 7, and 35 proteins had theoretical pI less than 7.

For stability of globular proteins at wide range of temperature, the higher aliphatic index is regarded as the positive factor for stability at high temperature. For better interaction of proteins with water molecules, very low GRAVY index is considered as helpful and was calculated through GRAVY calculator by the total sum of values of hydropathy for all amino acids, divided by the total length of the protein. Aliphatic and aromatic properties with average number of polar and nonpolar amino acids along with basic and acidic nature for all protein sequences were determined as shown in Supplementary Table 2.

### Functional annotation & pathway analysis of identified proteins

The proteins were classified based on biological processes, molecular functions and cellular components on the basis of Gene ontology annotations. At biological level, 20 processes were identified. Most of the proteins were found involving in small-molecule metabolic processes, cellular nitrogen compounds and biosynthetic processes. Twenty different molecular functions were identified out of which the largest cluster was found to be involved in ion-binding, followed by hydrolase and ligase activities. The cells had highest number, followed by plasma membrane and intracellular components (Figure 1). In addition, the pathways analysis showed that four proteins, namely WP_006344048.1, WP_006343878.1, WP_006343995.1 and WP_035395294.1, were involved in regulation of metabolism, biosynthesis and defense mechanisms. However, none of these four proteins shared homology with any of the host proteins. Three proteins (WP_006343878.1, WP_006343995.1 and WP_035395294.1) were involved in virulence.

### Potential drug target proteins in *C. abortus* as revealed by homology analysis

The nonhomologous proteins were inferred by homology search between pathogen protein sequences. The selection of the proteins which share sequence between pathogen and the host is not desirable. Hence, Basic Local Alignment Search Tool for proteins’ search of the C. *abortus* hypothetical proteins against the hosts, namely *Ovis aries* (sheep; taxid: 9940), *Capra aegagrus hircus* (goat; taxid: 9925), *Bos taurus* (bovine; taxid: 9913), *Bos grunniens* (yak; taxid: 30521), *Sus scrofa domesticus* (pig; taxid: 9825) and *Homo sapiens* (human; taxid: 9906) proteomes was carried out. As a result, only six hypothetical proteins of *C. abortus* showed homology with proteins present in host species (Table 3). These proteins were rejected or abandoned as targeting them in *C. abortus* may lead to cross-reactivity, auto-immune reactions and cytotoxicity in host.

### Structural analysis & MD simulation

Out of 45 proteins, the structural modeling of two proteins, in other words, WP_006344020.1 and WP_006344325.1 were successfully accomplished using comparative structural methods after selecting their suitable templates, namely 4QAS and 3DJA, respectively. The amino acid residues involved in the enzymatic activity of, first, WP_006344020.1 (phosphorylase) and second, WP_006344325.1 (chlamydial protease/proteasome-like activity factor [CPAF]) are shown in sticks (red color; Figure 2). To check the stability, compactness and structural behavior of modeled proteins, energy minimization and MD simulation were carried out. The proteins modeled were found to be stable as revealed from MD parameters including radius of gyration, the solvent accessible surface area and the root-mean-square fluctuations (RMSF; Figure 3). Validation of both models done by checking stereochemical quality of protein structure through the Ramachandran Plot analysis using PROCHECK showed that 93% residues were in allowed region in WP_006344020.1, and 95% residues were in allowed region of WP_006344325.1 (Supplementary Figures 1 & 2).

The comparison of the modeled structures of phosphorylase and CPAF homolog to their ortholog in *C. trachomatis* was carried out. It was found that they had similar structures as validated by superimposition of modeled structures and predicted ortholog (Supplementary Figure 3).

## Discussion

*Chlamydia* is an important pathogen with potential risks for humans and livestock species including poultry and other birds [11,48]. Studies have shown that *C. pecorum* is intestinal endemic pathogen in cattle, whereas other species like *C. pneuminiae, C. psittaci* and *C. gallinacea* were involved in systemic (uterine, blood and milk) infections [49].

Whole-genome sequence analysis of *C. abortus* has revealed highly variable protein families, including transmembrane head/inc and polymorphic membrane proteins and secretion systems [11]. It is important to investigate hypothetical proteins for deciphering their functions in pathogenic microorganisms to identify suitable drug targets. Predicting the functions of hypothetical proteins through bioinformatics methods is a quicker and preliminary approach, whereas the classical wet lab approaches such as enrichment and isolation, gene cloning and understanding functions at system level are expensive and protracted [50,51]. Through *in silico* approaches, the genes or proteins can be predicted initially and validated afterward by molecular biological methods.

Different *Chlamydia* species are already sequenced [9,16,48,52]. The structural analysis of proteins can boost up their predicted functions. By using homology-modeling methods, we could predict structures of two hypothetical proteins. Functional residues found in structures were analyzed by structural alignments for fully validation of predicted functions during sequence analysis. The protein sequences with expectation value less than 0.0001 were considered as homologous proteins [36]. The virulence factors of the 45 hypothetical proteins of *C. abortus* were identified as effective targets for drug discovery, as also reported in earlier studies [37].

### Classification of identified proteins

Identification of signature sequences and protein families has enabled the researchers to classify hypothetical proteins into different categories such as binding proteins, transmembrane, outer membrane proteins, enzymes, defense, secretory and signaling proteins. There were 14 hypothetical proteins in present study with their possible roles in nucleic acid-binding, and metal-binding processes. Some proteins were predicted for their possible role in DNA replication and recovery in case of DNA damage or genotoxicity. In addition, the proteins were also found to act as ribosomal proteins S3, transcription factors (e.g., NusA_K), and post-transcriptional modifiers of mRNA in stressed environments.

For defense against Gram-negative bacteria under stressed situations, the outer membrane proteins play a vital role. Five outer membrane proteins were predicted based on their pattern of alternating hydrophobic amino acids similar to porins present in outer membrane of bacteria.

A total of nine hypothetical proteins were predicted as enzymes, such as transferases, ATP synthases and GTPases. The secretory proteins (e.g., hormones, enzymes, antimicrobial peptides and toxins) are actively transported across the cell membrane. A total of five proteins were recognized as secretory proteins, one belonging to CesT family, and four to type-III secretory system. Protein secretory system type-III is used by bacteria to deliver their effector proteins into cells, which leads to modulation of host cellular functions [53,54]. Signal proteins have a precious role in several mechanisms including growth and immune systems. Almost all processes such as membrane fusion, transportation of ions, enzymatic activities, defense systems depend on signaling mechanisms. In addition, a total of ten proteins related to cellular signaling were identified. The earlier studies have speculated that cellular signaling proteins could be targeted for evolving novel therapeutic interventions against pathogenic *C. abortus* [55].

### Proteins involved in various metabolic pathways

A total of four proteins (WP_006344048.1, WP_006343878.1, WP_006343995.1 and WP_035395294.1) with various functions were identified. The protein WP_006344048.1 was identified as DNA polymerase III, with delta subunit having KEGG-id K02340 (EC: 2.7.7.7). It is required for assembly of the processivity factor β(2) onto primed DNA in the DNA polymerase III holoenzyme-catalyzed reaction. The delta subunit is also known as HolA. It has role in purine and pyrimidine metabolisms. It also catalyzes DNA-template-direct extension of the 3′-end of DNA strand, by one nucleotide at a time, hence plays a role in DNA mismatch repair. The protein WP_006343878.1 was annotated as arabinose-5-phosphate isomerase with KEGG-id K06041 (EC: 5.3.1.13). This enzyme has role in catalysis and synthesis of 3-deoxy-D-manno-octulosonate, which is a component of bacterial lipopolysaccharides. It constitutes outer bacterial membrane known as endotoxin and is referred to as lipid A. It is identified as pathogen-associated molecule by the host immune cells. The protein WP_006343995.1 was recognized as lipoate-protein ligase A with KEGG-id K03800 (EC: 2.7.7.63). This protein has role in regulating lipoic acid metabolism and is important for functioning of key enzymes involved in oxidative metabolism, dehydrogenases and transferases. The protein WP_035395294.1 was identified as substrate transport protein and acts as ABC transporters of ferric siderophores and metal ions such as Mn^2+^, Fe^3+^, Cu^2+^ and/or Zn^2+^ with KEGG-id K11707. The ligand-binding site is formed in the interface between two globular domains linked by a single helix. These may act as efflux pumps and helpful for bacterial defense mechanisms against drugs.

### Potential drug targets obtained as nonhomologous proteins in *C. abortus*

It is important to develop alternative strategies to cope with pathogenic microorganisms and preventing humans and animals from infections [56–58]. 45 nonhomologous proteins exclusively present in *C. abortus* have been found in this study. Six hypothetical proteins showed homology with the proteins present in various hosts. The 45 nonhomologous hypothetical proteins when subjected to virulence factors analysis revealed that 31 hypothetical proteins possessed virulence (Table 3). Evidently, a variety of toxins are produced by the pathogens to withstand the host immune system [59]. Furthermore, location of a protein in the cell is important in view of its interaction with other proteins or drug molecule targets. For instance, the cytoplasmic proteins act as promising drug targets while membrane proteins may be used as vaccine targets.

### Structural analysis of functional proteins

Structures of two proteins were finally predicted using comparative structural methods by selecting suitable templates. Protein WP_006344020.1 was recognized as Phosphorylase superfamily protein, having role in synthesis of quinones (menaquinone or ubiquinone), which are lipid-soluble electron carriers essential for cellular respiration in bacteria [60]. Although certain bacteria, for example, *Escherichia coli*, utilize different quinones depending on oxygen availability, many Gram-negative and most Gram-positive bacteria rely on menaquinone as the sole electron-carrier system [61].

This protein includes side chain of catalytic triad residue Asp-176, and main chain atoms Phe-83, Gly-85 and Tyr-139. This is found within a highly conserved region of prototypical 5′-methylthioadenosine nucleosidases and has consonance with some earlier reports [62]. Protein WP_006344325.1 was identified as CPAF that is responsible for degrading host molecules and plays a major role in chlamydial pathogenesis. The 3DJA was selected as template for prediction of structure. The highly conserved residues are Ser499 and His105 in CPAF, used for catalytic activity are well superimposed with the His97 and Ser488. On the other hand, auto-inhibition of CPAF is with internal inhibitory segment. Recognition of this peptide by the active site is dominated by two regions of contacts. One primarily involves hydrophobic contacts of the N-terminal rigid coil against the core domain of CPAF. The Met264 satisfactorily fits into the hydrophobic pocket formed by Val378, Cys500, Gly525 and F527 at template center which is similar in protein WP_006344325.1 as Met252 that fits into pocket formed by Val367, Cys489, Gly514 and Phe516. Additionally, a pair of main-chain hydrogen bonds is developed between Met252 and Gly367. Vander Waal contacts of three bulky residues, Phe256, Trp257 and Tyr264 in template, are similar to Phe268, Trp269 and Tyr276 in CPAF, with their respective neighboring residues which dominate interactions of the other binding region [63].

### Structural stability of proteins as validated using MD simulations

For a protein to be biologically active, it should be more stable to a unique globular conformation or native state. The factors such as amino acid sequence, folding, host cell strain, post-translational modifications, expression and purification conditions determine the protein stability. In this study, all the modeled proteins showed consistent structural behavior during their MD analysis. There were no abrupt fluctuations in the root-mean-square deviations and radius of gyration with the time evolution of trajectories of MD simulations. Moreover, the protein structure obtained had more compactness toward the end of MD. The RMSF analysis showed less variations and evenness in most of the amino acid residues. A steadiness in solvent accessible area was monitored which served as an evidence that predicted proteins would be stable in aqueous phase. The proteins did not show unusual folding/unfolding patterns during the simulations indicating that the proteins were stable. The overall analysis, in other words, radius of gyration, the solvent accessible area and RMSF analysis together validated the stability of the proteins.

Hypothetical protein WP_006344325.1 was identified as *Chlamydia* protease CPAF, unique to *Chlamydia*, degrades host molecules and assists the bacterium adapt host environment. Earlier, this protein was reported as the virulence factor in *Chlamydia* pathogenesis [63]. Our study also corroborated the same, and no homology was observed with any of the proteins in different hosts. Some of the proteins were annotated hypothetical in *Chlamydia*, for example, CT398(cdsZ). Later, the experimental work provided the evidence that the protein CT398(cdsZ) interacted with σ(54) (RpoN)-holoenzyme and the type-III secretion export apparatus in *C. trachomatis* [64]. Hypothetical protein CT263, with structural similarity to 5′-methylthioadenosine nucleosidase enzymes, supports the evidence that menaquinone synthesis in *Chlamydiaceae* is mediated through futalosine pathway [62]. A chromosomally encoded hypothetical protein TC0668 was found to serve as an important chromosome-encoded genitourinary pathogenicity factor in *C. muridarum* [65].

## Conclusion

Although hypothetical proteins are pseudogene, the hypothetical protein database possesses considerable amount of information. The analysis of unannotated data of *Chlamydia* has provided valuable inferences. The identified domains, virulence factor as well as tertiary structures of the proteins were validated by energy minimization and MD simulation. The *in silico* inferences could assist in computer-aided drug designing or vaccines to curtail the *C. abortus*.

## Future perspective

In future, the clinical microbiology and infection epidemiology will depend much on rapid molecular testing of pathogens and reliable microbiological diagnostics. Clinicians and medical practitioners will have to process and interpret the data obtained from sequencing technologies that are wholly different from conventional microbiological methods.

*Chlamydia* is an important infectious intracellular pathogen of humans and animals. Asymptomatic or paucisymptomatic infection that remains undetected, and therefore untreated for a prolonged duration, can lead to health problems including pelvic inflammatory disease, miscarriage in pregnant women and infertility. Many aspects of chlamydiology such as epidemiology, taxonomy and evolution, biodiversity, diagnosis and treatments are of concern to clinicians and readers.

Abundance of some *Chlamydia* species in poultry and exotic avian species reveals epidemiological importance of wild birds as potential reservoirs of the pathogen. The persons like poultry breeders, veterinarians, women involved in poultry gutting should take strict preventive measures against *Chlamydia* infections. The chlamydial infections are manageable in humans, but endemic in animals due to nonavailability of timely and effective antichlamydial treatments. Although hypothetical proteins are not linked to documented genes, their annotation may reveal novel biomolecules and biochemical pathways. The proteins investigated in the study are conserved in various *Chlamydia* species. Notably, the bioinformatics, computational biology and chemical engineering could lead to invention of novel metabolites with therapeutic potential from the microorganisms. The hypothetical proteins reported herein are envisioned to be novel targets for developing drugs to curtail chlamydial infections. However, the inferences should be validated by standard molecular biological methods.

**Table 1.** **List of bioinformatics programs used for functional analysis of hypothetical proteins in *Chlamydia abortus*.**

**Sl. No.**	**Programs**	**Prediction**	**Ref.**
1	NCBI-CDD	Protein-conserved domain	[18]
2	INTERPROSCAN	Protein domain	[19]
3	SVM-Prot	Protein functional family	[20]
4	Pfam	Protein family	[21]
5	SCOP-Superfamily	SCOP domains in proteins	[22]
6	EMBOOS PepStat	Statistics for protein	[25]
7	TMHMM	Transmembrane α-helices	[26]
8	HMMTOP	Transmembrane α-helices	[27]
9	SOSUI-GramN	Subcellular localization	[28]
10	CELLO	Subcellular localization	[29,30]
11	SignalP 4.1	Signal peptide cleavage site	[31]
12	CELLO2GO	Gene ontology	[32]
13	KEGG	Pathway analysis of proteins	[33]
14	Phyre2	Protein homology modeling	[40]
15	SAVES	Checking protein structure for molecular stereochemical quality, residues parameters, nonbonded interactions, model compatibility, macromolecular volume and the Ramachandran plot	[41–45]
16	GROMACS 5.0	Energy minimization and molecular dynamics of protein	[46]
17	PYMOL	Visualization of tertiary structure of proteins	[47]
18	BLAST	Similarity search between biological sequences	[34]

**Table 2.** **List of sequence-based analysis of 51 hypothetical proteins, gene IDs and some of their specific functions in *Chlamydia abortus*.**

**Sl. No.**	**Protein ID**	**Gene ID**	**Protein function**
1.	WP_006344227.1	493388305	DNase and RNase activity
2.	WP_006342697.1	493386499	RNA-binding domain
3.	WP_006343813.1	493387621	Involved in binding peptidoglycans
4.	WP_035395401.1	737414395	Plays roles in distinct ADP–ribose pathway
5.	WP_006344461.1	493388269	Regulates protein phosphorylation
6.	WP_006343888.1	493387696	Periplasmic-binding protein
7.	WP_006344021.1	493387829	Periplasmic-binding protein
8.	WP_006344141.1	493387949	Binds and stabilizes newly synthesized polypeptides
9.	WP_006343819.1	493387627	Stress response protein
10.	WP_006344147.1	493387955	Nucleic-acid-binding protein
11.	WP_006344255.1	493388063	Plays role in regulation of enzymes and changes the subcellular localization of signaling pathway components
12.	WP_006343878.1	493387686	Involved in CMP-Kdo biosynthesis pathway
13.	WP_006344558.1	493388367	TIM barrel proteins
14.	WP_006343973.1	493387781	DNA-binding proteins
15.	WP_006344427.1	493388235	Role in cell motility, the Ras pathway, vesicle trafficking and secretion, immune cell activation and apoptosis
16.	WP_006343829.1	493387637	P-53-associated protein
17.	WP_006344570.1	493388379	DNA double-strand break repair
18.	WP_006343995.1	493387803	Co-enzyme metabolism
19.	WP_006344048.1	493387856	DNA polymerase III
20.	WP_006344566.1	493388375	Catalyzes addition of l-glutamate
21.	WP_035395338.1	737414331	Type-1 glutamine amidotransferase (GATase1)-like domain
22.	WP_006344020.1	493387828	Catalytic and methylthioadenosine nucleosidase activity
23.	WP_006344334.1	493388142	Involved in bacterioruberin synthesis
24.	WP_006343689.1	493387496	Rhodanese homology
25.	WP_006343852.1	493387660	Ribokinase activity and carbohydrate phosphorylation
26.	WP_006343738.1	493387545	Involved in coupling of ATP degradation to H^+^ translocation
27.	WP_006343847.1	493387655	Involved in structural integrity
28.	WP_006343848.1	493387656	Involved in structural integrity
29.	WP_006343935.1	493387743	ChlamPMP_M superfamily
30.	WP_006343849.1	493387657	Forms disulfide bonds to provide rigidity to the cell wall
31.	WP_006344111.1	493387919	OmpH superfamily (characterizes as a molecular chaperon that interacts with unfolded proteins)
32.	WP_006343706.1	493387513	Tri chaperone protein like
33.	WP_006343695.1	493387502	Type III secretion system
34.	WP_006343696.1	493387503	Type III secretion system
35.	WP_006344485.1	493388293	Type III secretion system
36.	WP_006344486.1	493388294	Type III secretion system
37.	WP_006344325.1	493388133	Promotes the release of all-trans retinol
38.	WP_006344533.1	493388342	Architecture and deduce catalytic mechanism of PP2C phosphatase
39.	WP_006344534.1	493388343	Architecture and deduce catalytic mechanism of PP2C phosphatase
40.	WP_006343947.1	493387755	Function in fertility regulation
41.	WP_006343725.1	493387532	TPR domain (mitochondrial import protein)
42.	WP_006344218.1	493388026	Involved in protein–protein interactions, chaperons, cell cycle and transcription
43.	WP_035395294.1	737414287	Function in the ABC transport of ferric siderophores and metal ions such as Mn^2+^, Fe^3+^, Cu^2+^ and/or Zn^2+^
44.	WP_006344406.1	493388214	Alkaline shock protein
45.	WP_006344365.1	493388173	Shows anti-apoptotic effect and role in pathogen resistance
46.	WP_006343744.1	493387552	Globin-like protein (plays various roles in all three kingdoms of life)
47.	WP_006344477.1	493388285	Plays vital roles in the ISC and NIF systems of Fe–S protein maturation
48.	WP_006344274.1	493388082	Required for PSII to be fully operation *in vivo*
49.	WP_006343831.1	493387639	Metal-dependent hydrolase
50.	WP_006344578.1	493388387	Nucleotide transport and metabolism
51.	WP_006344167.1	493387975	Protein act as a virulence factor

ABC: ATP-binding cassette; CMP-Kdo: Cytidine 5-monophospho-3-deoxy-d-octulosonic acid; ISC: Iron-sulphur cluster; NIF: Nitrogen fixation; TIM: Triosephosphate isomerase; TPR: Tetratricopeptide repeat.

**Table 3.** **List of hypothetical proteins of *Chlamydia abortus* that were showing sequence homology with selected six host proteins.**

**Sl. No.**	**Protein ID**	**Gene ID**	**Sequence homology with host proteomes**						**Virulence factor**
			**Sheep**	**Goat**	**Bovine**	**Yak**	**Pig**	**Humans**	
1.	WP_006344227.1	493388305	×	×	×	×	×	×	Yes
2.	WP_006342697.1	493386499	×	×	×	×	×	×	Yes
3.	WP_006343813.1	493387621	×	×	×	×	×	×	×
4.	WP_035395401.1	737414395	×	×	×	×	×	×	×
5.	WP_006344461.1	493388269	×	×	×	×	×	×	×
6.	WP_006343888.1	493387696	×	×	×	×	×	×	Yes
7.	WP_006344021.1	493387829	×	×	×	×	×	×	×
8.	WP_006344141.1	493387949	×	×	×	×	×	×	×
9.	WP_006343819.1	493387627	×	×	×	×	×	×	×
10.	WP_006344147.1	493387955	×	×	×	×	×	×	Yes
11.	WP_006344255.1	493388063	×	×	×	×	×	×	×
12.	WP_006343878.1	493387686	×	×	×	×	×	×	Yes
13.	WP_006344558.1	493388367	Yes	Yes	Yes	×	×	Yes	Rejected
14.	WP_006343973.1	493387781	×	×	×	×	×	×	×
15.	WP_006344427.1	493388235	×	×	×	×	×	×	Yes
16.	WP_006343829.1	493387637	×	Yes	Yes	×	×	Yes	Rejected
17.	WP_006344570.1	493388379	×	×	×	×	×	×	×
18.	WP_006343995.1	493387803	×	×	×	×	×	×	Yes
19.	WP_006344048.1	493387856	×	×	×	×	×	×	×
20.	WP_006344566.1	493388375	×	×	×	×	×	×	Yes
21.	WP_035395338.1	737414331	×	×	×	×	×	×	Yes
22.	WP_006344020.1	493387828	×	×	×	×	×	×	Yes
23.	WP_006344334.1	493388142	×	×	×	×	×	×	Yes
24.	WP_006343689.1	493387496	Yes	Yes	Yes	×	×	Yes	Rejected
25.	WP_006343852.1	493387660	×	×	×	×	×	×	Yes
26.	WP_006343738.1	493387545	×	×	×	×	×	×	Yes
27.	WP_006343847.1	493387655	×	×	×	×	×	×	Yes
28.	WP_006343848.1	493387656	×	×	×	×	×	×	Yes
29.	WP_006343935.1	493387743	×	×	×	×	×	×	Yes
30.	WP_006343849.1	493387657	×	×	×	×	×	×	Yes
31.	WP_006344111.1	493387919	×	×	×	×	×	×	Yes
32.	WP_006343706.1	493387513	×	×	×	×	×	×	Yes
33.	WP_006343695.1	493387502	×	×	×	×	×	×	Yes
34.	WP_006343696.1	493387503	×	×	×	×	×	×	Yes
35.	WP_006344485.1	493388293	×	×	×	×	×	×	Yes
36.	WP_006344486.1	493388294	×	×	×	×	×	×	×
37.	WP_006344325.1	493388133	×	×	×	×	×	×	Yes
38.	WP_006344533.1	493388342	×	×	×	×	×	×	Yes
39.	WP_006344534.1	493388343	×	×	×	×	×	×	×
40.	WP_006343947.1	493387755	Yes	Yes	Yes	×	×	Yes	Rejected
41.	WP_006343725.1	493387532	Yes	Yes	Yes	×	×	Yes	Rejected
42.	WP_006344218.1	493388026	×	×	×	×	×	×	Yes
43.	WP_035395294.1	737414287	×	×	×	×	×	×	Yes
44.	WP_006344406.1	493388214	×	×	×	×	×	×	Yes
45.	WP_006344365.1	493388173	×	×	×	×	×	×	Yes
46.	WP_006343744.1	493387552	×	×	×	×	×	×	Yes
47.	WP_006344477.1	493388285	×	Yes	×	×	×	×	Rejected
48.	WP_006344274.1	493388082	×	×	×	×	×	×	×
49.	WP_006343831.1	493387639	×	×	×	×	×	×	Yes
50.	WP_006344578.1	493388387	×	×	×	×	×	×	Yes
51.	WP_006344167.1	493387975	×	×	×	×	×	×	×

The virulence factors were checked in the nonhomologous proteins. Six proteins showing homology with proteins present in host were rejected because they could also be targeted.

**Executive summary**

**Background**

*Chlamydia abortus* is an important, obligate intracellular pathogen of humans, livestock species, pet and companion animals, poultry and wild birds.

**Methodology**

Data extraction & identification of conserved signatures:
  The *C. abortus* LLG with genome reference number NZ_CM001168.1 at NCBI Genome database served as data source;
  Functional signature sequences of hypothetical proteins were identified by Web-based bioinformatics tools.
Characterization & localization of proteins with conserved signatures:
  Theoretical isoelectric point (pI), molecular weight and subcellular localization of proteins, and grand average hydropathicity were determined.
Functional categorization & pathway analysis of proteins:
  The functional roles of hypothetical proteins, and gene ontology were predicted.
Homology analysis of protein sequences with host proteins & virulence factor analysis:
  The virulence factor of nonhomologous proteins was identified.
Structural analysis & model evaluation of functionally identified proteins:
  For residues in most favored region, the Ramachandran plot was analyzed in PROCHECK at SAVES.

**Results & discussion**

Data extraction & analysis of hypothetical proteins:
  51 proteins were predicted for their role in metabolic pathways, defense, binding and transporting other biomolecules;
  No discrepancy between the official annotation and analysis by our methodology was observed.
Localization of hypothetical proteins with conserved signatures:
  13 proteins were predicted to have transmembranic helices, 32 were cytoplasmic, three as extracellular, five as innermembranic, eight as outermembranic and three proteins as periplasmic;
  Only single peptide is transmembrane.
Physiochemical characterization of proteins with functional domain:
  Of the 51 proteins with conserved domain and some specific functions, 16 proteins had theoretical pI ≥7, and 35 proteins had theoretical pI <7;
  Aliphatic and aromatic properties of polar and nonpolar amino acids along with basic and acidic nature were determined.
Functional annotation & pathway analysis of identified proteins:
  Most of the proteins involved in small-molecule metabolic processes, cellular nitrogen compound and biosynthetic processes;
  The proteins WP_006344048.1, WP_006343878.1, WP_006343995.1 and WP_035395294.1 were involved in regulation of metabolism, biosynthesis and defense;
  Three proteins (WP_006343878.1, WP_006343995.1 and WP_035395294.1) were involved in virulence. Six hypothetical proteins had homology with proteins in host species.
Structural analysis & molecular dynamics simulation:
  The structural modeling of two proteins, first, WP_006344020.1 (phosphorylase) and second, WP_006344325.1 (chlamydial protease/proteasome-like activity factor) were achieved. The proteins were stable as revealed from molecular dynamics simulation.

**Future perspective**

The hypothetical proteins are envisioned as probable targets for developing drugs against pathogen.

## References

[1] Entrican G, Wheelhouse NM (2006). Immunity in the female sheep reproductive tract. *Vet. Res.*.

[2] Longbottom D, Livingstone M, Maley S (2013). Intranasal infection with *Chlamydia abortus* induces dose-dependent latency and abortion in sheep. *PLoS ONE*.

[3] Gomes MJP, Wald VB, Machado RD (2001). Isolamento de *Chlamydia psittaci* em touros com vesiculite seminal no Rio Grande Do Sul. *A Hora Veterinaria*.

[4] Rekiki A, Sidi-Boumedine K, Souriau A (2002). Isolation and characterisation of local strains of *Chlamydophila abortus* (*Chlamydia psittaci* serotype 1) from Tunisia. *Vet. Res.*.

[5] Pospischil A, Thoma R, Hilbe M (2002). [Abortion in humans caused by *Chlamydophila abortus* (*Chlamydia psittaci* serovar 1)]. *Schweiz. Arch. Tierheilkd.*.

[6] Baud D, Regan L, Greub G (2008). Emerging role of *Chlamydia* and *Chlamydia*-like organisms in adverse pregnancy outcomes. *Curr. Opin. Infect. Dis.*.

[7] Chen Q, Gong X, Zheng F (2014). Seroprevalence of *Chlamydophila abortus* infection in yaks (*Bos grunniens*) in Qinghai, China. *Trop. Anim. Health Prod.*.

[8] Hoffmann K, Schott F, Donati M (2015). Prevalence of chlamydial infections in fattening pigs and their influencing factors. *PLoS ONE*.

[9] Azuma Y, Hirakawa H, Yamashita A (2006). Genome sequence of the cat pathogen, *Chlamydophila felis*. *DNA Res.*.

[10] Kang YH, Cong W, Qin SY (2016). First report of *Toxoplasma gondii, Dirofilaria immitis*, and *Chlamydia felis* infection in stray and companion cats in North-Eastern and Eastern China. *Vector Borne Zoonotic Dis.*.

[11] Laroucau K, Aaziz R, Meurice L (2015). Outbreak of psittacosis in a group of women exposed to *Chlamydia psittaci*-infected chickens. *Euro Surveill.*.

[12] Essig A, Longbottom D (2015). *Chlamydia abortus*: new aspects of infectious abortion in sheep and potential risk for pregnant women. *Curr. Clin. Microbiol. Rep.*.

[13] Everett KD, Bush RM, Andersen AA (1999). Emended description of the order Chlamydiales, proposal of *Parachlamydiaceae* fam. nov. and *Simkaniaceae* fam. nov., each containing one monotypic genus, revised taxonomy of the family *Chlamydiaceae*, including a new genus and five new species, and standards for the identification of organisms. *Int. J. Syst. Bacteriol.*.

[14] Sachse K, Bavoil PM, Kaltenboeck B (2015). Emendation of the family *Chlamydiaceae*: proposal of a single genus, *Chlamydia*, to include all currently recognized species. *Syst. Appl. Microbiol.*.

[15] Pannekoek Y, Qin QL, Zhang YZ (2016). A Genus delineation of Chlamydiales by analysis of the percentage of conserved proteins (POCP) justifies the reunifying of the genera *Chlamydia* and *Chlamydophila* into one single genus *Chlamydia*. *Pathog. Dis.*.

[16] Sait M, Clark EM, Wheelhouse N (2011). Genome sequence of the *Chlamydophila abortus* variant strain LLG. *J. Bacteriol.*.

[17] NCBI: Genome http://www.ncbi.nlm.nih.gov/genome.

[18] Marchler-Bauer A, Derbyshire MK, Gonzales NR (2015). CDD: NCBI's conserved domain database. *Nucleic Acids Res.*.

[19] Quevillon E, Silventoinen V, Pillai S (2005). InterProScan: protein domains identifier. *Nucleic Acids Res.*.

[20] Cai CZ, Han LY, Ji ZL (2003). SVM-Prot: web-based support vector machine software for functional classification of a protein from its primary sequence. *Nucleic Acids Res.*.

[21] Finn RD, Bateman A, Clements J (2014). Pfam: the protein families database. *Nucleic Acids Res.*.

[22] Gough J, Chothia C (2002). SUPERFAMILY: HMMs representing all proteins of known structure. SCOP sequence searches, alignments and genome assignments. *Nucleic Acids Res.*.

[23] Gasteiger E, Hoogland C, Gattiker A, Walker JM (2005). Protein identification and analysis tools on the ExPASy server. *The Proteomics Protocols Handbook*.

[24] GRAVY Calculator http://www.gravy-calculator.de/.

[25] Rice P, Longden I, Bleasby A (2000). EMBOSS: the European Molecular Biology Open Software Suite. *Trends Genet.*.

[26] Krogh A, Larsson B, von Heijne G (2001). Predicting transmembrane protein topology with a hidden Markov model: application to complete genomes. *J. Mol. Biol.*.

[27] Tusnády GE, Simon I (2001). The HMMTOP transmembrane topology prediction server. *Bioinformatics*.

[28] Imai K, Asakawa N, Tsuji T (2008). SOSUI-GramN: high performance prediction for sub-cellular localization of proteins in gram-negative bacteria. *Bioinformation*.

[29] Yu CS, Lin CJ, Hwang JK (2004). Predicting subcellular localization of proteins for Gram-negative bacteria by support vector machines based on n-peptide compositions. *Protein Sci.*.

[30] Yu CS, Chen YC, Lu CH (2006). Prediction of protein subcellular localization. *Proteins*.

[31] Petersen TN, Brunak S, von Heijne G (2011). SignalP 4.0: discriminating signal peptides from transmembrane regions. *Nat. Methods*.

[32] Yu CS, Cheng CW, Su WC (2014). CELLO2GO: a web server for protein subCELlular LOcalization prediction with functional gene ontology annotation. *PLoS ONE*.

[33] Kanehisa M, Goto S, Sato Y (2014). Data, information, knowledge and principle: back to metabolism in KEGG. *Nucleic Acids Res.*.

[34] Altschul SF, Madden TL, Schäffer AA (1997). Gapped BLAST and PSI-BLAST: a new generation of protein database search programs. *Nucleic Acids Res.*.

[35] Protein BLAST http://www.blast.ncbi.nlm.nih.gov/Blast.cgi?PAGE=Proteins.

[36] Sarangi AN, Aggarwal R, Rahman Q (2009). Subtractive genomics approach for *in silico* identification and characterization of novel drug targets in *Neisseria meningitides* Serogroup B. *J. Comput. Sci. Syst. Biol.*.

[37] Baron C, Coombes B (2007). Targeting bacterial secretion systems: benefits of disarmament in the microcosm. *Infect. Disord. Drug Targets*.

[38] Saha S, Raghava GP (2006). VICMpred: an SVM-based method for the prediction of functional proteins of Gram-negative bacteria using amino acid patterns and composition. *Genomics Proteomics Bioinformatics*.

[39] Saha S, Raghava GP (2007). BTXpred: prediction of bacterial toxins. *In Silico Biol.*.

[40] Kelley LA, Mezulis S, Yates CM (2015). The Phyre2 web portal for protein modeling, prediction and analysis. *Nat. Protoc.*.

[41] Lüthy R, Bowie JU, Eisenberg D (1992). Assessment of protein models with three-dimensional profiles. *Nature*.

[42] Colovos C, Yeates TO (1993). Verification of protein structures: patterns of nonbonded atomic interactions. *Protein Sci.*.

[43] Hooft RW, Vriend G, Sander C (1996). Errors in protein structures. *Nature*.

[44] Pontius J, Richelle J, Wodak SJ (1996). Deviations from standard atomic volumes as a quality measure for protein crystal structures. *J. Mol. Biol.*.

[45] Laskowski RA, MacArthur MW, Moss DS (1993). PROCHECK: a program to check the stereochemical quality of protein structures. *J. Appl. Cryst.*.

[46] Berendsen HJC, van der Spoel D, van Drunen R (1995). GROMACS: a message-passing parallel molecular dynamics implementation. *Computer Phys. Commun.*.

[47] DeLano WL The PyMOL molecular graphics system. http://www.pymol.org/.

[48] Hölzer M, Laroucau K, Creasy HH (2016). Whole-genome sequence of *Chlamydia gallinacea* type strain 08–1274/3. *Genome Announc.*.

[49] Li J, Guo W, Kaltenboeck B (2016). *Chlamydia pecorum* is the endemic intestinal species in cattle while *C. gallinacea, C. psittaci* and *C. pneumoniae* associate with sporadic systemic infection. *Vet. Microbiol.*.

[50] Roche DB, McGuffin LJ (2016). Toolbox for protein structure prediction. *Methods Mol. Biol.*.

[51] Mazandu GK, Mulder NJ (2012). Function prediction and analysis of mycobacterium tuberculosis hypothetical proteins. *Int. J. Mol. Sci.*.

[52] Thomson NR, Yeats C, Bell K (2005). The *Chlamydophila abortus* genome sequence reveals an array of variable proteins that contribute to interspecies variation. *Genome Res.*.

[53] Hueck CJ (1998). Type III protein secretion systems in bacterial pathogens of animals and plants. *Microbiol. Mol. Biol. Rev.*.

[54] Galán JE, Collmer A (1999). Type III secretion machines: bacterial devices for protein delivery into host cells. *Science*.

[55] Choudhary C, Weinert BT, Nishida Y (2014). The growing landscape of lysine acetylation links metabolism and cell signalling. *Nat. Rev. Mol. Cell Biol.*.

[56] Kumar M, Yadav A, Verma V (2016). Bioengineered probiotics as a new hope for health and diseases: potential and prospects: an overview. *Future Microbiol.*.

[57] Singh B, Mal G, Bharti D (2013). Probiotics in female reproductive health: paradigms, prospects and challenges. *Curr. Women's Health Rev.*.

[58] Singh B, Mal G, Bissi L (2016). The Holy Grail of designer probiotics: the probiotics with multiple health benefits. *J. Gastrointest. Digestive System*.

[59] Aktories K, Barbieri JT (2005). Bacterial cytotoxins: targeting eukaryotic switches. *Nat. Rev. Microbiol.*.

[60] Kurosu M, Begari E (2010). Vitamin K2 in electron transport system: are enzymes involved in vitamin K2 biosynthesis promising drug targets?. *Molecules*.

[61] Unden G, Bongaerts J (1997). Alternative respiratory pathways of *Escherichia coli*: energetics and transcriptional regulation in response to electron acceptors. *Biochim. Biophys. Acta*.

[62] Barta ML, Thomas K, Yuan H (2014). Structural and biochemical characterization of *Chlamydia trachomatis* hypothetical protein CT263 supports that menaquinone synthesis occurs through the futalosine pathway. *J. Biol. Chem.*.

[63] Huang Z, Feng Y, Chen D (2008). Structural basis for activation and inhibition of the secreted chlamydia protease CPAF. *Cell Host Microbe*.

[64] Barta ML, Battaile KP, Lovell S (2015). Hypothetical protein CT398 (CdsZ) interacts with σ(54) (RpoN)-holoenzyme and the type III secretion export apparatus in *Chlamydia trachomatis*. *Protein Sci.*.

[65] Conrad TA, Gong S, Yang Z (2015). The chromosome-encoded hypothetical protein TC0668 is an upper genital tract pathogenicity factor of *Chlamydia muridarum*. *Infect. Immun.*.

