# Corrigendum

**DOI:** 10.4155/fsoa-2016-0066c1

**Published:** 2017-10-05

Following publication of the Research Article by Gagandeep Singh, Dixit Sharma, Vikram Singh, Jyoti Rani, Francessco Marotta, Manoj Kumar, Gorakh Mal, Birbal Singh, titled ‘*In silico* functional elucidation of uncharacterized proteins of *Chlamydia abortus* strain LLG’, which appeared in the March 2017 issue of *Future Science OA* 3[1], FSO169 [2017]; doi: 10.4155/fsoa-2016-0066), it has been brought to our attention that Dixit Sharma's affiliation was incorrect and should have read:

ICAR-Indian Veterinary Research Institute, Regional Station, Palampur-176061, India

The authors and editors of *Future Science OA* would like to sincerely apologise for any confusion or inconvenience this may have caused our readers.

