# Supplementary material for: In silico functional elucidation of uncharacterized proteins of Chlamydia abortus strain LLG
Source: Future Sci OA. 2017 Jan 24;3(1):FSO169. doi: 10.4155/fsoa-2016-0066 (PMC5351547; doi:10.4155/fsoa-2016-0066)
Supplement: Supplementary file 4 [file fsoa-03-169-s4.docx]

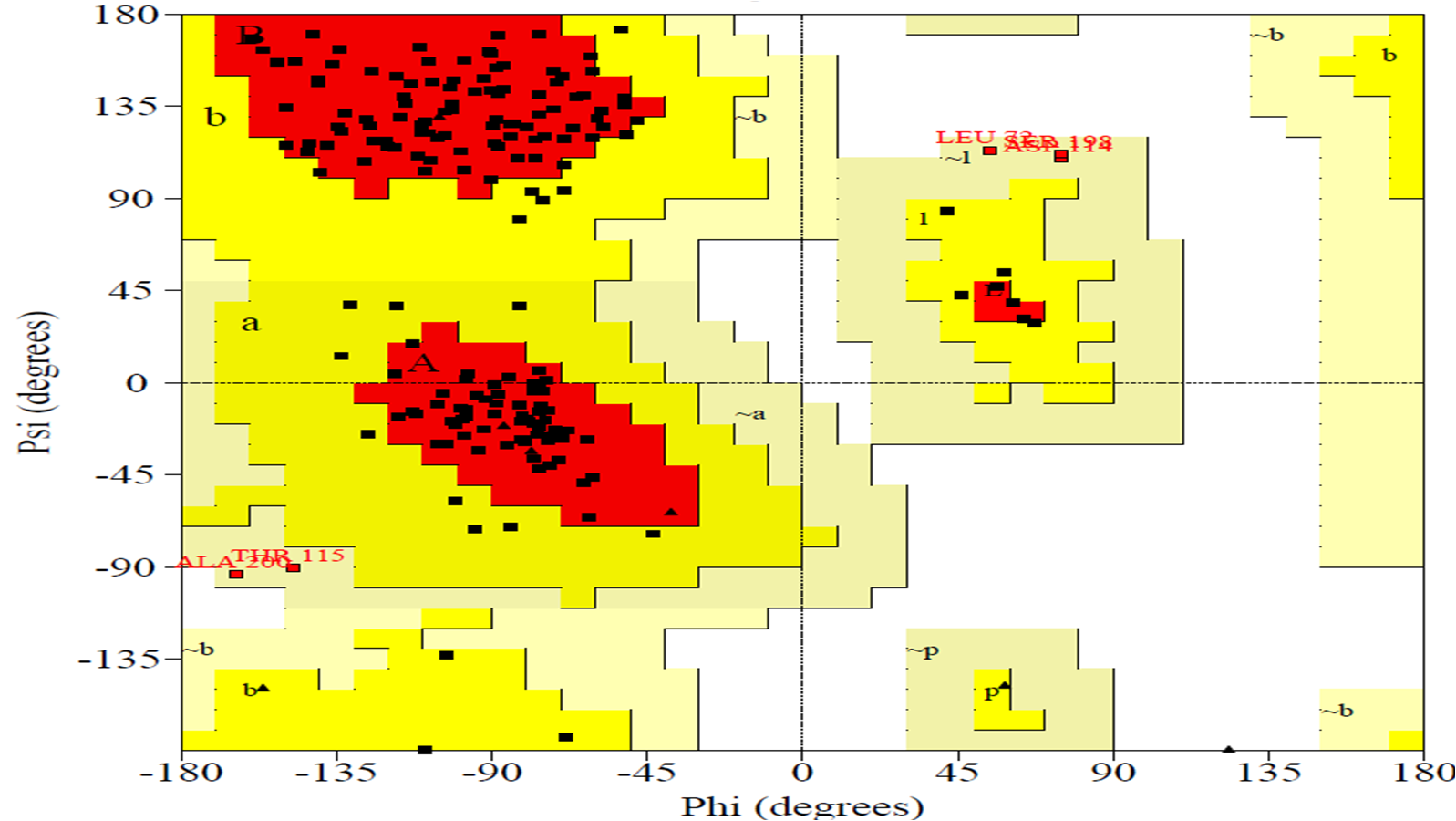


**Supplementary Figure 1**. Ramachandran Plot for Protein WP_006344020.1. The description is included in text.


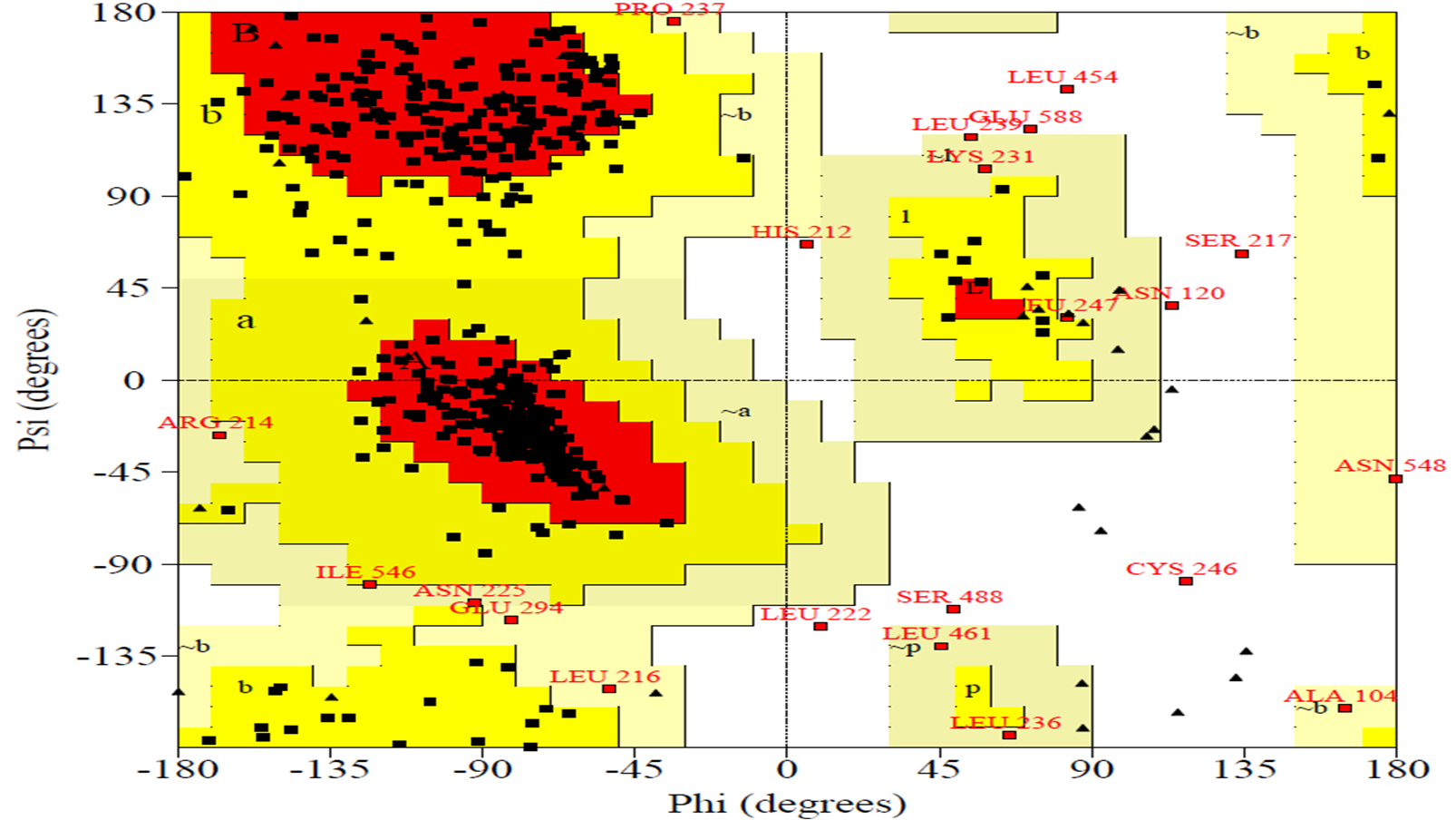


**Supplementary Figure 2.** Ramachandran Plot for Protein WP_006344325.1. The description is included in text.


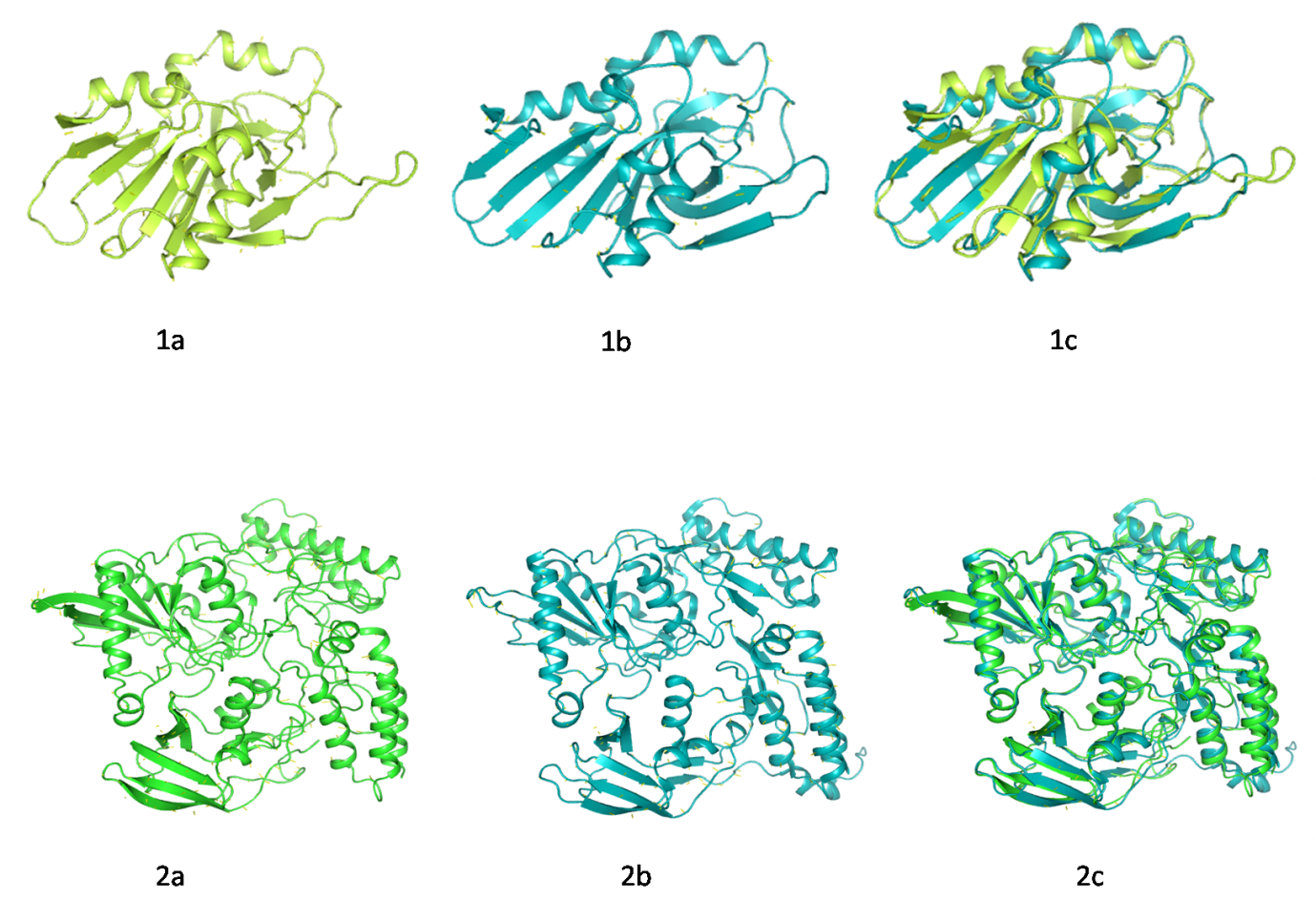


**Supplementary Figure 3.** Comparative structural analysis of WP_0063440201.1 (Phosphorylase) and WP_006344325.1 (CPAF) with their orthologue in *C. trachomatis* showed that the structural features i.e. α-helix and β-sheet present along the whole sequence is conserved.

**1a.** WP_006344020.1 (Phosphorylase), **1b.** Orthologue of WP_006344020.1 in *C. trachomatis* (4QAS), **1c.** Superimposed structure of protein WP_006344020.1 with orthologue (4QAS) in *C. trachomatis*.

**2a.** WP_006344325.1 (CPAF), **2b.** Orthologue of WP_006344325.1 in C. trachomatis (4QAS), **2c.** Superimposed structure of protein WP_006344325.1 with orthologue (3DJA) in *C. trachomatis*.
